# *catena*-Poly[[(1,10-phenanthroline)zinc]-μ-3-[3-(carboxyl­atometh­oxy)phen­yl]acrylato]

**DOI:** 10.1107/S1600536812024610

**Published:** 2012-06-13

**Authors:** Ling Chen

**Affiliations:** a School of Pharmacy and Material Engineering, Jinhua College of Vocation and Technology, Jinhua, Zhejiang 321017, People’s Republic of China

## Abstract

The asymmetric unit of the title compound, [Zn(C_11_H_8_O_5_)(C_12_H_8_N_2_)]_*n*_, is composed of a Zn^II^ ion and 3-[3-(carboxyl­atometh­oxy)phen­yl]acrylate and 1,10-phenanthroline ligands. The Zn^II^ ion adopts a distorted square-pyramidal ZnN_2_O_3_ coordination. The bridging mode of the dianion leads to the formation of zigzag chains parallel to [010]. Intermolecular π–π stacking inter­actions [centroid–centroid distance of 3.5716 (12) Å] lead to the formation of a two-dimensional network parallel to (001).

## Related literature

For background to inorganic-organic hybrid materials, see: Fujita *et al.* (1994 ▶) and for their applications and topological structures, see: Comotti *et al.* (2008 ▶); Hong *et al.* (2006 ▶); Moulton & Zaworotko (2001 ▶); Swiegers & Malefeste (2000 ▶); Kaes *et al.* (2000 ▶).
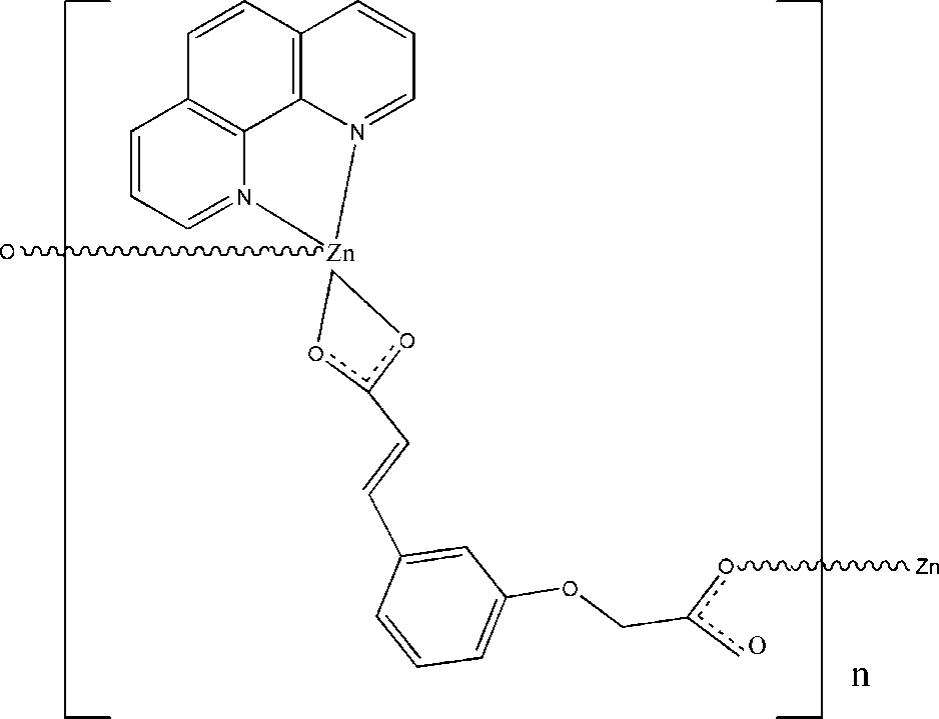


## Experimental

### 

#### Crystal data

[Zn(C_11_H_8_O_5_)(C_12_H_8_N_2_)]
*M* *_r_* = 465.75
Monoclinic, 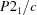

*a* = 10.2744 (4) Å
*b* = 14.9979 (6) Å
*c* = 15.9060 (6) Å
β = 127.347 (2)°
*V* = 1948.51 (13) Å^3^
*Z* = 4
Mo *K*α radiation
μ = 1.30 mm^−1^
*T* = 296 K
0.46 × 0.42 × 0.26 mm

#### Data collection

Bruker APEXII area-detector diffractometer
Absorption correction: multi-scan (*SADABS*; Sheldrick, 1996 ▶) *T*_min_ = 0.56, *T*_max_ = 0.71
26006 measured reflections
3430 independent reflections
2980 reflections with *I* > 2σ(*I*)
*R*_int_ = 0.022

#### Refinement

*R*[*F* ^2^ > 2σ(*F* ^2^)] = 0.023
*wR*(*F* ^2^) = 0.070
*S* = 1.02
3430 reflections
280 parameters
H-atom parameters constrained
Δρ_max_ = 0.22 e Å^−3^
Δρ_min_ = −0.35 e Å^−3^

### 

Data collection: *APEX2* (Bruker, 2006 ▶); cell refinement: *SAINT* (Bruker, 2006 ▶); data reduction: *SAINT*; program(s) used to solve structure: *SHELXS97* (Sheldrick, 2008 ▶); program(s) used to refine structure: *SHELXL97* (Sheldrick, 2008 ▶); molecular graphics: *DIAMOND* (Brandenburg, 1999 ▶); software used to prepare material for publication: *SHELXTL* (Sheldrick, 2008 ▶.

## Supplementary Material

Crystal structure: contains datablock(s) I, global. DOI: 10.1107/S1600536812024610/bg2464sup1.cif

Structure factors: contains datablock(s) I. DOI: 10.1107/S1600536812024610/bg2464Isup2.hkl

Additional supplementary materials:  crystallographic information; 3D view; checkCIF report

## Figures and Tables

**Fig. 1.**
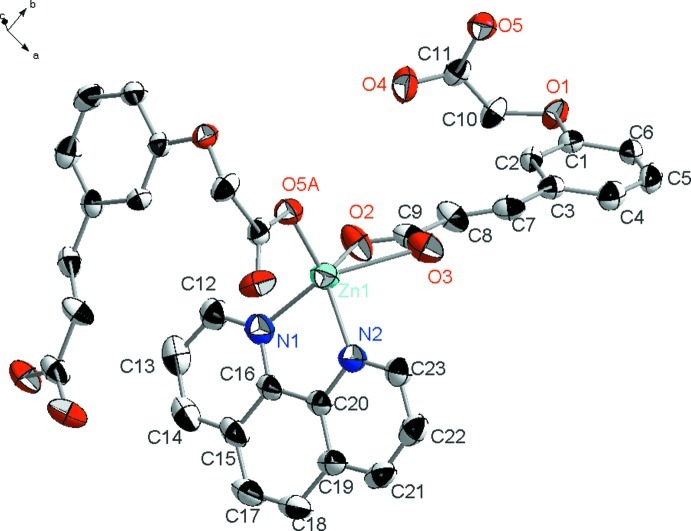
The molecular structure of the title compound. Displacement ellipsoids are drawn at the 50% probability level. [Symmetry code:(A) -x - 1,y - 1/2,-z + 1/2]

**Fig. 2.**
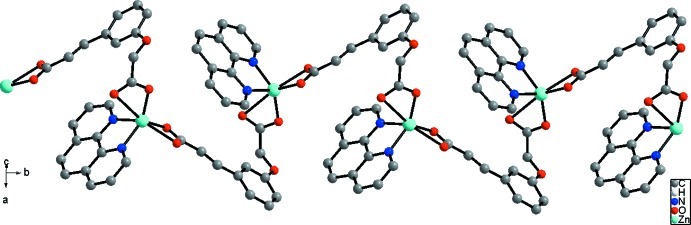
The one-dimensional chain structure in the title compound along the b axis.

**Fig. 3.**
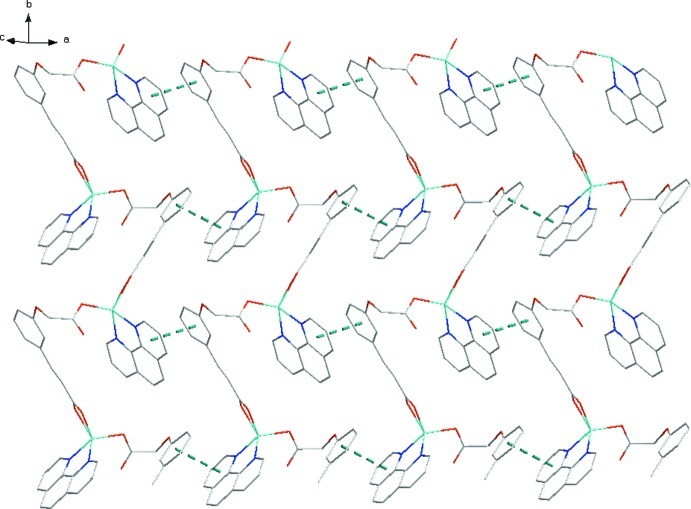
View of the two-dimensional supramolecular network connected by π-π stacking interactions.

## supplementary crystallographic information

### Comment

In recent decades, inorganic-organic hybrid materials such as coordination complexes have attracted plenty of attention (Fujita *et al.* (1994)) due to the fact that they might have potential applications as functional solid materials in adsorption, catalysis, and ion exchange (Comotti *et al.* (2008), Hong *et al.* (2006)), at the same time that they usually present intriguing topological structures (Moulton *et al.*(2001), Swiegers *et al.*(2000),Kaes *et al.*(2000)). Herein we report one such inorganic-organic hybrid compound, [Zn *L* (phen), where *L* is 3-carboxymethoxy phenyl acrylate (C_11_H_8_O_5_) and phen is 1,10-phenanthroline(C_12_H_8_N_2_)].

As shown in Figure 1, the strucure contains one Zn^II^ ion coordinated by two N atoms from a chelating phen and two O atoms from a chelating carboxylato group from one *L* ligand, defining the base of a distorted square pyramidal coordination; the apical site is occupied by a third O from the reamining carboxylato group of another *L* ligand which thus behaves in a µ_2_κ^3^ bridging chelating mode, forming a one-dimensional zigzag chain which extends along the *b* axis (Figure 2).

In this structure, the benzene ring from a *L* ligand and an adjacent six-membered heterocycle ring of phen are nearly parallel (dihedral angle: 4.83 (10)°), affording a face-to-face intermolecular π-π stacking with an intercentroid distance of 3.5716 (12) Å. Intermolecular π-π stacking interactions lead to the formation of a two-dimensional network (Figure 3).

### Experimental

A mixture of ZnCl_2_(0.136 g,1 mmol),3-carboxymethoxy phenycl acrylic acid(0.2220 g,1 mmol) and 1,10-phenanthroline(0.0991 g,0.5 mmol) was dissolved in a 20 mL EtOH/H_2_O(*v*/*v*,1:9). Then, the pH value was adjusted to 7 through the use of a 2 mol/*L* NaOH solution. The mixture was then sealed in a 25 mL stainless steel reactor and heated to 433 K for 3 days. Then the reactant mixture was cooled to room temperature at the rate of 5 degrees per hour. Evaporation of the resulting solution for a few days afforded colorless crystals of title compound.

### Refinement

The carbon-bound H-atoms were positioned geometrically and included in the refinement using a riding model [C—H 0.93 Å *U*_iso_(H) = 1.2*U*_eq_(C)].

### Crystal data

**Table:**

[Zn(C_11_H_8_O_5_)(C_12_H_8_N_2_)]	*F*(000) = 952
*M**_r_* = 465.75	*D*_x_ = 1.588 Mg m^−^^3^
Monoclinic, *P*2_1_/*c*	Mo *K*α radiation, λ = 0.71073 Å
Hall symbol: -P 2ybc	Cell parameters from 9958 reflections
*a* = 10.2744 (4) Å	θ = 2.1–25.0°
*b* = 14.9979 (6) Å	µ = 1.30 mm^−^^1^
*c* = 15.9060 (6) Å	*T* = 296 K
β = 127.347 (2)°	Prism, colourless
*V* = 1948.51 (13) Å^3^	0.46 × 0.42 × 0.26 mm
*Z* = 4	

### Data collection

**Table:**

Bruker APEXII area-detector diffractometer	3430 independent reflections
Radiation source: fine-focus sealed tube	2980 reflections with *I* > 2σ(*I*)
Graphite monochromator	*R*_int_ = 0.022
ω scans	θ_max_ = 25.0°, θ_min_ = 2.1°
Absorption correction: multi-scan (*SADABS*; Sheldrick, 1996)	*h* = −12→12
*T*_min_ = 0.56, *T*_max_ = 0.71	*k* = −17→17
26006 measured reflections	*l* = −18→18

### Refinement

**Table:**

Refinement on *F*^2^	Primary atom site location: structure-invariant direct methods
Least-squares matrix: full	Secondary atom site location: difference Fourier map
*R*[*F*^2^ > 2σ(*F*^2^)] = 0.023	Hydrogen site location: inferred from neighbouring sites
*wR*(*F*^2^) = 0.070	H-atom parameters constrained
*S* = 1.02	*w* = 1/[σ^2^(*F*_o_^2^) + (0.0426*P*)^2^ + 0.5701*P*] where *P* = (*F*_o_^2^ + 2*F*_c_^2^)/3
3430 reflections	(Δ/σ)_max_ = 0.001
280 parameters	Δρ_max_ = 0.22 e Å^−^^3^
0 restraints	Δρ_min_ = −0.35 e Å^−^^3^

### Special details

**Table:**

Geometry. All e.s.d.'s (except the e.s.d. in the dihedral angle between two l.s. planes) are estimated using the full covariance matrix. The cell e.s.d.'s are taken into account individually in the estimation of e.s.d.'s in distances, angles and torsion angles; correlations between e.s.d.'s in cell parameters are only used when they are defined by crystal symmetry. An approximate (isotropic) treatment of cell e.s.d.'s is used for estimating e.s.d.'s involving l.s. planes.
Refinement. Refinement of *F*^2^ against ALL reflections. The weighted *R*-factor *wR* and goodness of fit *S* are based on *F*^2^, conventional *R*-factors *R* are based on *F*, with *F* set to zero for negative *F*^2^. The threshold expression of *F*^2^ > σ(*F*^2^) is used only for calculating *R*-factors(gt) *etc*. and is not relevant to the choice of reflections for refinement. *R*-factors based on *F*^2^ are statistically about twice as large as those based on *F*, and *R*- factors based on ALL data will be even larger.

### Fractional atomic coordinates and isotropic or equivalent isotropic displacement parameters (Å^2^)

**Table:**

	*x*	*y*	*z*	*U*_iso_*/*U*_eq_	
Zn1	−0.30637 (2)	−0.253939 (12)	0.368035 (16)	0.03728 (9)	
O1	−0.33062 (15)	0.25494 (8)	0.04670 (10)	0.0419 (3)	
O2	−0.36123 (19)	−0.15717 (9)	0.26359 (11)	0.0591 (4)	
O3	−0.13946 (19)	−0.12441 (9)	0.42086 (11)	0.0567 (4)	
O4	−0.66285 (17)	0.13485 (10)	0.00573 (12)	0.0591 (4)	
O5	−0.56836 (17)	0.26740 (9)	0.07863 (12)	0.0482 (3)	
N1	−0.41141 (18)	−0.35805 (9)	0.25550 (12)	0.0408 (3)	
N2	−0.10093 (17)	−0.33092 (9)	0.42815 (12)	0.0373 (3)	
C1	−0.20554 (19)	0.21845 (11)	0.14171 (12)	0.0320 (3)	
C2	−0.22189 (19)	0.14486 (10)	0.18669 (13)	0.0335 (4)	
H2A	−0.3230	0.1168	0.1518	0.040*	
C3	−0.0880 (2)	0.11214 (11)	0.28402 (13)	0.0353 (4)	
C4	0.0613 (2)	0.15579 (13)	0.33550 (14)	0.0442 (4)	
H4A	0.1511	0.1361	0.4015	0.053*	
C5	0.0764 (2)	0.22891 (13)	0.28838 (15)	0.0449 (4)	
H5A	0.1773	0.2571	0.3227	0.054*	
C6	−0.0547 (2)	0.26020 (11)	0.19240 (15)	0.0377 (4)	
H6A	−0.0429	0.3089	0.1614	0.045*	
C7	−0.1050 (2)	0.03192 (12)	0.32975 (14)	0.0412 (4)	
H7A	−0.0254	0.0223	0.4017	0.049*	
C8	−0.2224 (3)	−0.02701 (12)	0.27791 (15)	0.0473 (5)	
H8A	−0.3011	−0.0182	0.2056	0.057*	
C9	−0.2398 (3)	−0.10691 (12)	0.32551 (15)	0.0457 (4)	
C10	−0.4780 (2)	0.20433 (14)	−0.01510 (13)	0.0433 (4)	
H10A	−0.4506	0.1438	−0.0206	0.052*	
H10B	−0.5465	0.2291	−0.0859	0.052*	
C11	−0.5770 (2)	0.20077 (12)	0.02659 (13)	0.0386 (4)	
C12	−0.5648 (2)	−0.37041 (13)	0.17056 (16)	0.0511 (5)	
H12A	−0.6440	−0.3295	0.1562	0.061*	
C13	−0.6122 (3)	−0.44236 (15)	0.10171 (17)	0.0602 (6)	
H13A	−0.7209	−0.4489	0.0429	0.072*	
C14	−0.4975 (3)	−0.50280 (14)	0.12172 (17)	0.0583 (5)	
H14A	−0.5279	−0.5515	0.0771	0.070*	
C15	−0.3334 (3)	−0.49139 (12)	0.20977 (16)	0.0486 (5)	
C16	−0.2961 (2)	−0.41737 (11)	0.27537 (14)	0.0382 (4)	
C17	−0.2041 (3)	−0.55048 (13)	0.23654 (19)	0.0583 (6)	
H17A	−0.2280	−0.5997	0.1938	0.070*	
C18	−0.0487 (3)	−0.53644 (13)	0.32207 (19)	0.0564 (6)	
H18A	0.0330	−0.5756	0.3367	0.068*	
C19	−0.0068 (2)	−0.46234 (12)	0.39092 (16)	0.0447 (4)	
C20	−0.1308 (2)	−0.40297 (11)	0.36709 (14)	0.0369 (4)	
C21	0.1518 (2)	−0.44575 (13)	0.48294 (17)	0.0506 (5)	
H21A	0.2373	−0.4837	0.5020	0.061*	
C22	0.1806 (2)	−0.37366 (14)	0.54458 (16)	0.0503 (5)	
H22A	0.2852	−0.3625	0.6061	0.060*	
C23	0.0510 (2)	−0.31708 (12)	0.51406 (15)	0.0443 (4)	
H23A	0.0720	−0.2675	0.5557	0.053*	

### Atomic displacement parameters (Å^2^)

**Table:**

	*U*^11^	*U*^22^	*U*^33^	*U*^12^	*U*^13^	*U*^23^
Zn1	0.04366 (15)	0.02930 (13)	0.04777 (15)	0.00345 (7)	0.03236 (12)	0.00291 (8)
O1	0.0319 (6)	0.0483 (8)	0.0439 (7)	0.0026 (5)	0.0221 (6)	0.0141 (5)
O2	0.0862 (11)	0.0392 (7)	0.0518 (8)	−0.0111 (7)	0.0418 (8)	0.0017 (6)
O3	0.0772 (9)	0.0435 (8)	0.0504 (8)	0.0091 (7)	0.0393 (8)	0.0136 (6)
O4	0.0534 (8)	0.0638 (9)	0.0682 (9)	−0.0194 (7)	0.0411 (7)	−0.0084 (7)
O5	0.0560 (8)	0.0423 (7)	0.0640 (9)	0.0012 (6)	0.0456 (8)	0.0041 (6)
N1	0.0480 (9)	0.0336 (7)	0.0481 (9)	−0.0012 (6)	0.0330 (8)	0.0024 (6)
N2	0.0435 (8)	0.0309 (7)	0.0492 (9)	0.0005 (6)	0.0342 (8)	0.0025 (6)
C1	0.0317 (9)	0.0319 (8)	0.0352 (9)	0.0016 (6)	0.0217 (8)	−0.0012 (7)
C2	0.0284 (8)	0.0327 (8)	0.0368 (9)	−0.0036 (6)	0.0184 (7)	−0.0016 (7)
C3	0.0363 (9)	0.0358 (9)	0.0343 (9)	0.0028 (7)	0.0217 (8)	−0.0015 (7)
C4	0.0329 (9)	0.0543 (11)	0.0327 (9)	0.0013 (8)	0.0132 (8)	−0.0043 (8)
C5	0.0327 (9)	0.0508 (10)	0.0453 (11)	−0.0119 (8)	0.0205 (9)	−0.0131 (8)
C6	0.0390 (10)	0.0334 (9)	0.0477 (11)	−0.0064 (7)	0.0300 (9)	−0.0061 (7)
C7	0.0447 (10)	0.0411 (10)	0.0382 (9)	0.0121 (8)	0.0254 (8)	0.0091 (8)
C8	0.0663 (12)	0.0362 (9)	0.0389 (10)	0.0006 (9)	0.0316 (10)	0.0053 (8)
C9	0.0702 (13)	0.0329 (9)	0.0505 (12)	0.0086 (9)	0.0452 (11)	0.0040 (8)
C10	0.0311 (9)	0.0620 (12)	0.0338 (9)	0.0001 (8)	0.0182 (8)	0.0049 (8)
C11	0.0306 (8)	0.0460 (10)	0.0341 (9)	0.0058 (8)	0.0170 (8)	0.0110 (8)
C12	0.0493 (11)	0.0481 (11)	0.0543 (12)	−0.0018 (9)	0.0305 (10)	0.0058 (9)
C13	0.0628 (13)	0.0589 (13)	0.0528 (12)	−0.0191 (11)	0.0319 (11)	−0.0051 (10)
C14	0.0806 (15)	0.0440 (11)	0.0616 (13)	−0.0192 (11)	0.0491 (13)	−0.0127 (10)
C15	0.0738 (14)	0.0333 (9)	0.0597 (12)	−0.0089 (9)	0.0514 (12)	−0.0034 (8)
C16	0.0541 (11)	0.0273 (8)	0.0509 (10)	−0.0009 (7)	0.0410 (9)	0.0031 (7)
C17	0.0897 (17)	0.0348 (10)	0.0808 (16)	−0.0005 (10)	0.0675 (15)	−0.0061 (10)
C18	0.0836 (16)	0.0334 (10)	0.0880 (16)	0.0105 (10)	0.0708 (15)	0.0066 (10)
C19	0.0605 (12)	0.0332 (9)	0.0658 (12)	0.0073 (8)	0.0516 (11)	0.0114 (8)
C20	0.0501 (10)	0.0272 (8)	0.0509 (10)	0.0006 (7)	0.0398 (9)	0.0049 (7)
C21	0.0528 (11)	0.0457 (11)	0.0734 (14)	0.0138 (9)	0.0488 (11)	0.0213 (10)
C22	0.0436 (10)	0.0563 (12)	0.0571 (12)	0.0029 (9)	0.0336 (10)	0.0148 (10)
C23	0.0491 (11)	0.0414 (10)	0.0514 (11)	−0.0023 (8)	0.0352 (10)	0.0019 (8)

### Geometric parameters (Å, º)

**Table:**

Zn1—O5^i^	1.9502 (13)	C7—C8	1.307 (3)
Zn1—O2	2.0119 (13)	C7—H7A	0.9300
Zn1—N2	2.0626 (14)	C8—C9	1.485 (2)
Zn1—N1	2.1126 (15)	C8—H8A	0.9300
Zn1—O3	2.3818 (15)	C10—C11	1.515 (2)
Zn1—C9	2.5197 (19)	C10—H10A	0.9700
O1—C1	1.371 (2)	C10—H10B	0.9700
O1—C10	1.425 (2)	C12—C13	1.398 (3)
O2—C9	1.266 (2)	C12—H12A	0.9300
O3—C9	1.239 (2)	C13—C14	1.363 (3)
O4—C11	1.229 (2)	C13—H13A	0.9300
O5—C11	1.266 (2)	C14—C15	1.403 (3)
O5—Zn1^ii^	1.9502 (13)	C14—H14A	0.9300
N1—C12	1.327 (2)	C15—C16	1.408 (2)
N1—C16	1.357 (2)	C15—C17	1.430 (3)
N2—C23	1.326 (2)	C16—C20	1.433 (3)
N2—C20	1.358 (2)	C17—C18	1.345 (3)
C1—C2	1.380 (2)	C17—H17A	0.9300
C1—C6	1.389 (2)	C18—C19	1.431 (3)
C2—C3	1.395 (2)	C18—H18A	0.9300
C2—H2A	0.9300	C19—C21	1.402 (3)
C3—C4	1.388 (2)	C19—C20	1.405 (2)
C3—C7	1.470 (2)	C21—C22	1.366 (3)
C4—C5	1.389 (3)	C21—H21A	0.9300
C4—H4A	0.9300	C22—C23	1.394 (3)
C5—C6	1.367 (3)	C22—H22A	0.9300
C5—H5A	0.9300	C23—H23A	0.9300
C6—H6A	0.9300		
			
O5^i^—Zn1—O2	108.22 (6)	O3—C9—C8	121.79 (19)
O5^i^—Zn1—N2	130.55 (6)	O2—C9—C8	116.76 (17)
O2—Zn1—N2	118.81 (6)	O3—C9—Zn1	69.26 (10)
O5^i^—Zn1—N1	110.93 (6)	O2—C9—Zn1	52.27 (9)
O2—Zn1—N1	95.18 (6)	C8—C9—Zn1	168.31 (14)
N2—Zn1—N1	80.34 (6)	O1—C10—C11	115.57 (15)
O5^i^—Zn1—O3	103.69 (5)	O1—C10—H10A	108.4
O2—Zn1—O3	58.93 (5)	C11—C10—H10A	108.4
N2—Zn1—O3	88.72 (5)	O1—C10—H10B	108.4
N1—Zn1—O3	142.20 (5)	C11—C10—H10B	108.4
O5^i^—Zn1—C9	109.35 (6)	H10A—C10—H10B	107.4
O2—Zn1—C9	29.86 (6)	O4—C11—O5	124.43 (17)
N2—Zn1—C9	104.25 (6)	O4—C11—C10	118.21 (17)
N1—Zn1—C9	120.11 (6)	O5—C11—C10	117.33 (16)
O3—Zn1—C9	29.10 (6)	N1—C12—C13	122.7 (2)
C1—O1—C10	116.18 (13)	N1—C12—H12A	118.7
C9—O2—Zn1	97.87 (11)	C13—C12—H12A	118.7
C9—O3—Zn1	81.63 (12)	C14—C13—C12	119.3 (2)
C11—O5—Zn1^ii^	110.61 (11)	C14—C13—H13A	120.3
C12—N1—C16	118.40 (16)	C12—C13—H13A	120.3
C12—N1—Zn1	130.30 (13)	C13—C14—C15	119.82 (19)
C16—N1—Zn1	111.24 (12)	C13—C14—H14A	120.1
C23—N2—C20	118.40 (15)	C15—C14—H14A	120.1
C23—N2—Zn1	128.60 (12)	C14—C15—C16	117.25 (19)
C20—N2—Zn1	112.97 (11)	C14—C15—C17	123.94 (19)
O1—C1—C2	124.29 (14)	C16—C15—C17	118.80 (19)
O1—C1—C6	115.46 (15)	N1—C16—C15	122.52 (17)
C2—C1—C6	120.24 (15)	N1—C16—C20	117.81 (15)
C1—C2—C3	120.56 (15)	C15—C16—C20	119.67 (16)
C1—C2—H2A	119.7	C18—C17—C15	121.51 (19)
C3—C2—H2A	119.7	C18—C17—H17A	119.2
C4—C3—C2	118.79 (16)	C15—C17—H17A	119.2
C4—C3—C7	120.96 (16)	C17—C18—C19	121.12 (19)
C2—C3—C7	120.24 (15)	C17—C18—H18A	119.4
C3—C4—C5	119.93 (16)	C19—C18—H18A	119.4
C3—C4—H4A	120.0	C21—C19—C20	117.30 (17)
C5—C4—H4A	120.0	C21—C19—C18	123.79 (18)
C6—C5—C4	121.12 (17)	C20—C19—C18	118.90 (19)
C6—C5—H5A	119.4	N2—C20—C19	122.48 (17)
C4—C5—H5A	119.4	N2—C20—C16	117.53 (15)
C5—C6—C1	119.32 (17)	C19—C20—C16	119.99 (16)
C5—C6—H6A	120.3	C22—C21—C19	119.86 (17)
C1—C6—H6A	120.3	C22—C21—H21A	120.1
C8—C7—C3	125.61 (17)	C19—C21—H21A	120.1
C8—C7—H7A	117.2	C21—C22—C23	119.14 (19)
C3—C7—H7A	117.2	C21—C22—H22A	120.4
C7—C8—C9	125.05 (18)	C23—C22—H22A	120.4
C7—C8—H8A	117.5	N2—C23—C22	122.81 (18)
C9—C8—H8A	117.5	N2—C23—H23A	118.6
O3—C9—O2	121.44 (17)	C22—C23—H23A	118.6

Symmetry codes: (i) −*x*−1, *y*−1/2, −*z*+1/2; (ii) −*x*−1, *y*+1/2, −*z*+1/2.
